# Trans fatty acids – A risk factor for cardiovascular disease

**DOI:** 10.12669/pjms.301.4525

**Published:** 2014

**Authors:** Mohammad Perwaiz Iqbal

**Affiliations:** 1 Mohammad Perwaiz Iqbal, Department of Biological & Biomedical Sciences, Aga Khan University, Stadium Road, P.O. Box-3500, Karachi-74800, Pakistan.

**Keywords:** Cardiovascular disease, Coronary heart disease, Dietary fats, Trans fatty acid, South Asia

## Abstract

Trans fatty acids (TFA) are produced either by hydrogenation of unsaturated oils or by biohydrogenation in the stomach of ruminant animals. Vanaspati ghee and margarine have high contents of TFA. A number of studies have shown an association of TFA consumption and increased risk of cardiovascular disease (CVD). This increased risk is because TFA increase the ratio of LDL cholesterol to HDL cholesterol. Food and Agriculture Organization of the United Nations and World Health Organization have come up with the recommendation that the contents of TFA in human dietary fat should be reduced to less than 4%. There is high prevalence of CVD in Pakistan. High consumption of vanaspati ghee which contains 14.2-34.3% of TFA could be one of the factors for this increased burden of CVD in Pakistan. Consumption of dietary fat low in TFA would be helpful in reducing the risk of CVD in South Asia. Denmark by banning the sale of food items with TFA has brought down the number of deaths due to coronary heart disease by nearly 50% over a period of 20 years. Public awareness about the adverse effects of TFA on human health would be extremely important. Media can play a very effective role in educating the masses and advocating the policy for the sale of only low TFA food items.

***Literature sources:*** Google and US National Library of Medicine, National Institute of Health were the sources of papers cited in this review article.

## INTRODUCTION

***Trans fatty acids: ***Trans fatty acids (TFA) by definition are geometric isomers of monounsaturated and polyunsaturated fatty acids having at least one carbon-carbon double bond with hydrogens on opposite sides of the double bond (trans configuration).^1^

They arise either by partial hydrogenation of unsaturated oils (usually in the presence of nickel) or by biohydrogenation in the rumens of cows and sheep.^2^ Due to their ability to pack more tightly than other unsaturated fatty acids, they have a higher melting point (45C) than oleic acid and, therefore, would add granularity (semisolid form) to the hydrogenated oil.^3^ TFA arising due to biohydrogenation in stomach of ruminant animals are present in small amounts in dairy fat (milk, butter) and meat fat. These natural TFA include conjugated linoleic acid (CLA) and vaccenic acid.^3^

***Trans fatty acids in food items: ***Margarine, vanaspati ghee, bakery and frying fats and vegetable shortenings are obtained through industrial hydrogenation and contain significant amounts of TFA. Since their contents vary from one food item to another, it is difficult to estimate their consumption in various countries. However, in USA it is estimated to be 2-3 energy percent, while in some of the countries in Middle East and South Asia, it could be as high as 7 energy percent.^4^

In South Asian countries, vanaspati ghee is the major source of TFA. For example, in India, vanaspati used to contain as high as 40-50% TFA.^5^^,^^6^ In Iran, 33% of fatty acids in partially hydrogenated oils were TFA^7^; while in Pakistan TFA contents ranged from 14.2-34.3% in various vanaspati samples.^8^ Margarine is another major source of TFA. Hard-type margarine samples in Pakistan contained TFA in the range of 1.6-23.1%, while soft margarines had less than 4.1%.^8^

***Trans fatty acids and cardiovascular disease: ***As the adverse effects of dietary saturated fatty acids became known, TFA were considered to be a safe replacement of butter fat. However, in 1990 Mensink and Katan reported that TFA increased total and low-density lipoprotein (LDL) cholesterol and decreased the “good” high-density lipoprotein (HDL) cholesterol.^9^ Willet et al at Harvard University enunciated the hypothesis that intake of TFA increased the risk of coronary heart disease (CHD) by increasing the ratio of LDL cholesterol to HDL cholesterol.^10^

In 2006, a comprehensive review of a large number of TFA related studies indicated a strong association between consumption of TFA and CHD, concluding “On a per-calorie basis, trans fats appear to increase the risk of CHD more than any other micronutrient.^11^ A more conclusive evidence came from the Nurses’ Health Study in which CHD risk roughly doubled for each 2% increase in trans fat calories consumed instead of carbohydrate calories.^12^ On the basis of several such studies, it was concluded that there were no nutritional benefits of TFA and there were clear adverse effects especially increasing the risk of cardiovascular disease (CVD), therefore a prudent policy should be adopted to limit their consumption and contents in various food items.^13^

Food and Agriculture Organization (FAO) of the United Nations and World Health Organization came up with the recommendation that the contents of TFA in human dietary fats should be reduced to less than 4%.^14^ Denmark was the first country to introduce ban on the consumption of partially hydrogenated oils.^15^ It is generally believed that because of Danish Government’s efforts in reducing TFA consumption from 6 gm to 1 gm per day over a period of 2 decades, the deaths among Danes due to CHD have dropped by 50%.^16^

Regarding South Asia, it has been estimated that 39% of CHD events in Iran can be prevented by replacement of TFA with cis-unsaturated fats.^7^ Indian Government in an attempt to combat TFA related health problems plans to reduce TFA contents in vanaspati oil to 5% by the end of 2013.^5^ However, in Pakistan no such regulatory measures are in place regarding limiting the consumption of TFA. Due to alarmingly rising trend of CVD in Pakistan,^17^ it is imperative that reduction/removal of TFA from food items be regulated through legislation and a public awareness campaign about the risks associated with TFA consumption should be initiated by health-care professionals.

***Ruminant TFA:*** Ruminant TFA which are produced by biohydrogenation in stomach of ruminant animals include vaccenic acid and rumenic acid which is a conjugated linoleic acid isomer.^18^ Both isomers are present in dairy products. While industrial TFA have been clearly shown to be associated with the increased risk of CVD, there is some evidence of health benefits due to ruminant TFA.^19^ However, this area still needs further research.^20^

***How to reduce TFA in food products?***

1. Reformulation of food products is an effective way of reducing TFA. For example, in USA, trans fat contents were reduced to less than 0.5 gm per serving in 95% of the supermarket products analyzed and 80% of the restaurant products analyzed.^21^
2. *Fat interesterification*: Interesterified fat is a type of oil where the fatty acids have been moved from one triglyceride molecule to another. Interesterification of vegetable oils can replace an unsaturated fatty acid (such as oleic acid or linoleic acid) at the middle position (sn-2) of glycerol by a saturated fatty acid (stearic acid) with the help of catalysts or lipase enzyme.^22^ Majority of the long term studies did not suggest that interesterification of dietary fats have any adverse change in lipoprotein profile. However, more research is required in this field with focus on the effect of interesterified fat on inflammatory markers. ^23^
3. *Genetically modified fatty acids:* Using genetic engineering, fatty acids with desired characteristics can be obtained and then through reformulation the TFA contents of food products can be reduced.^24^

***Recommendations:***

There is an epidemic of CVD in Pakistan.^17^ It is seriously affecting the economy of the country.^25^ Adoption of healthy food items can be one of the major ways towards reducing the burden of CVD. TFA adverse effects on CVD are well established, therefore, their consumption should be brought to a minimum level by the following methods:

1. Substitution of natural plant oils containing lower percent of polyunsaturated fatty acids for vanaspati.
2. Edible oil industry should adopt alternative technologies to reduce TFA to at least WHO recommended levels (<4%).^14^
3. Frying has been found to result into the formation of TFA.^26^ Therefore; deep-fried food items are likely to contain high contents of TFA. Adoption of gentle-frying or steam-frying of food is going to reduce TFA formation in these food items. This would be an effective process of preparing healthy food in our homes. In the Cardiovascular Health Study, it has been shown that increased consumption of fried fish sandwiches was associated with increased risk of CVD.^27^ Therefore, increased intake of tuna and non-fried fish with presumably small amount of TFA would be a healthy approach.^28^
4. Repeated use of cooking oil is a common practice in Pakistan. TFA contents are already high in hydrogenated vegetable oils and these would increase further after repeated frying.^8^ Therefore, repeated use of cooking oil should be avoided.
5. Recent research has indicated that high intake of dairy saturated fat is associated with decreased risk of CVD, while higher intake of meat saturated fat has been found to be associated with greater CVD risk.^29^ Therefore; use of saturated fat from dairy sources instead of TFA containing oils is a healthy option.
6. Consumers’ awareness and education about the adverse effects of TFA is the most important step to avoid TFA rich food items. Media can play a major role in public advocacy.
7. Laws should be promulgated by the Government to introduce mandatory labeling for information of TFA and saturated fat content on packs of vanaspati oil, edible oil and other oil products. Without the Government’s resolve, it would be difficult to achieve the objective. Success of Danish Government in decreasing the number of deaths due to CHD by 50% over the past 20 years by reducing the intake of TFA among Danes from 6 gm to 1 gm per day is a shining example.^3^^,^^16^

In summary, a multipronged approach involving both public and private sectors would be needed to reduce the burden of CVD in South Asia through the adoption of healthy foods.
